# Pretreatment NPLH as a Potential Predictor of Pathologic Complete Response to Accelerated MVAC Neoadjuvant Chemotherapy in Muscle-Invasive Bladder Cancer: Comparison with NLR and PLR

**DOI:** 10.3390/cancers18132046

**Published:** 2026-06-24

**Authors:** Łukasz Kwinta, Kamil Konopka, Krzysztof Okoń, Mateusz Łobacz, Maciej Lubaś, Piotr Chłosta, Przemysław Dudek, Piotr J. Wysocki

**Affiliations:** 1 Oncology Department, Faculty of Medicine, Jagiellonian University Medical College, 31-501 Krakow, Poland; 2 Department of Oncology, University Hospital in Krakow, 31-501 Krakow, Poland; 3 Department of Pathomorphology, Faculty of Medicine, Jagiellonian University Medical College, 31-531 Krakow, Poland; 4 Department of Pathomorphology, University Hospital in Krakow, 30-688 Krakow, Poland; 5 Department of Urology, Faculty of Medicine, Jagiellonian University Medical College, 30-688 Krakow, Poland; 6 Clinical Department of Urology and Oncological Urology, University Hospital in Krakow, 30-688 Krakow, Poland; 7 Department of Urology, Medical University of Vienna, University Hospital Vienna, 1090 Vienna, Austria

**Keywords:** NPLH, neutrophil-to-platelet-hemoglobin-lymphocyte ratio, NLR, PLR, NPLH score, muscle-invasive bladder cancer, MIBC, neoadjuvant chemotherapy, pathologic complete response, biomarker, MVAC

## Abstract

**Background.** Accurate prediction of pathologic complete response (pCR) to neoadjuvant chemotherapy (NAC) in muscle-invasive urothelial bladder cancer (MIBC) remains an unmet clinical need. The neutrophil-to-platelet/hemoglobin-to-lymphocyte (NPLH) ratio, a composite hematologic index that reflects both systemic inflammation and nutritional oxygen-carrying capacity, has not been previously evaluated as a predictor of NAC response in this setting. **Methods.** We retrospectively analyzed 114 consecutive patients with MIBC (cT2–T4, N0–N3) who received accelerated MVAC (aMVAC) NAC followed by radical cystectomy at a single academic center. Pretreatment NPLH (calculated as [neutrophils × platelets]/[hemoglobin × lymphocytes]) was assessed as a predictor of pCR (ypT0N0) and tumor regression grade (TRG). Receiver operating characteristic (ROC) curve analysis, Mann–Whitney U test, and logistic regression were used. NPLH performance was compared to NLR and PLR. **Results.** pCR was achieved in 35 patients (30.7%). Median NPLH was significantly lower in pCR vs. non-pCR patients (33.9 [IQR 23.1–42.4] vs. 47.6 [IQR 30.7–90.4]; *p* = 0.0007). NPLH yielded an AUC of 0.700 (bootstrap 95% CI 0.596–0.794) for pCR prediction, numerically superior to NLR (AUC 0.645 [0.542–0.741]) and PLR (AUC 0.643 [0.533–0.747]); DeLong test: NPLH vs. NLR *p* = 0.079, NPLH vs. PLR *p* = 0.090. At the optimal cut-off of 44.5, NPLH demonstrated 80.0% sensitivity and 57.0% specificity. pCR rates declined progressively across NPLH quartiles: 48.3% (Q1) to 10.3% (Q4). On multivariate logistic regression, log-transformed NPLH was the only independent predictor of pCR (parsimonious model, OR 0.292, 95% CI 0.131–0.652; *p* = 0.003; EPV = 17.5). A positive correlation was observed between NPLH and TRG score (Spearman r = 0.284; *p* = 0.0022), with significant differences between TRG 1 and TRG 3 subgroups (*p* = 0.0036). **Conclusions.** Pretreatment NPLH is an independent predictor of pCR to aMVAC in MIBC and is numerically superior to NLR and PLR (DeLong *p* = 0.079). Consisting exclusively of standard complete blood count parameters, NPLH is readily available and inexpensive. This single-center exploratory study is hypothesis-generating and requires prospective external validation before clinical implementation.

## Simple Summary

Accurate prediction of pathologic complete response (pCR) to neoadjuvant chemotherapy (NAC) in muscle-invasive urothelial bladder cancer (MIBC) remains an unmet clinical need. We retrospectively analyzed 114 consecutive patients with MIBC who received accelerated MVAC (aMVAC) NAC followed by radical cystectomy. Pretreatment NPLH (calculated as [neutrophils × platelets]/[hemoglobin × lymphocytes]) was assessed as a predictor of pCR (ypT0N0) and tumor regression grade (TRG). Median NPLH was significantly lower in pCR vs. non-pCR patients (33.9 [IQR 23.1–42.4] vs. 47.6 [IQR 30.7–90.4]; *p* = 0.0007). At the optimal cut-off of 44.5, NPLH demonstrated 80.0% sensitivity and 57.0% specificity. On multivariate logistic regression, log-transformed NPLH was the only independent predictor of pCR in the parsimonious two-covariate model (OR 0.292, 95% CI 0.131–0.652; *p* = 0.003; EPV = 17.5). NPLH demonstrated numerically higher AUC (0.700 [95% CI 0.596–0.794]) than NLR (0.645 [0.542–0.741]) and PLR (0.643 [0.533–0.747]), though differences were not statistically significant (DeLong test: *p* = 0.079 and *p* = 0.090, respectively). In this exploratory analysis, high pretreatment NPLH was associated with reduced probability of pCR; however, given the modest AUC and data-derived threshold, these findings require external validation before clinical application.

## 1. Introduction

Muscle-invasive urothelial bladder cancer (MIBC) accounts for approximately 25% of all bladder cancer cases and remains associated with poor oncologic outcomes. When treated with surgery alone, the 5-year cancer-specific mortality exceeds 50% [1,2]. Cisplatin-based neoadjuvant chemotherapy (NAC) followed by radical cystectomy (RC) is currently the standard treatment approach. Although the overall survival benefit of NAC is modest (approximately 5–8%), patients achieving pathologic complete response (pCR; ypT0N0) derive substantial improvement in long-term outcomes [3,4]. Previous studies have demonstrated that patients with pCR may achieve a median overall survival (OS) exceeding 77 months, compared with 46 months among non-responders [4].

However, only 30–40% of patients achieve pCR after NAC. Consequently, a large proportion of patients undergo RC and experience chemotherapy-related toxicity without significant oncologic benefit. Current NAC regimens include dose-dense or accelerated MVAC protocols, while gemcitabine/cisplatin-based combinations with durvalumab are increasingly used in patients with impaired renal function [5,6,7]. At the same time, novel perioperative strategies such as enfortumab vedotin combined with pembrolizumab are showing promising early results in MIBC [8,9,10]. As treatment options expand, identifying patients unlikely to respond to platinum-based NAC becomes increasingly important. Reliable prediction of non-response could support treatment individualization and help avoid unnecessary toxicity.

Several biomarkers predicting NAC response in MIBC have been investigated, including molecular subtypes, alterations in DNA damage response genes, and imaging-based parameters [11,12,13]. However, many of these methods require specialized testing or advanced equipment that may not be routinely available. In contrast, systemic inflammatory markers derived from routine complete blood count (CBC) are inexpensive and widely accessible. Among them, the neutrophil-to-lymphocyte ratio (NLR) and platelet-to-lymphocyte ratio (PLR) have been associated with tumor-related inflammation and immune dysregulation across multiple malignancies [14,15]. In MIBC, elevated pretreatment NLR has been associated with worse prognosis following RC; however, its predictive value for NAC response remains unknown [16,17].

The NPLH index (neutrophils × platelets/hemoglobin × lymphocytes) is a composite hematologic parameter integrating inflammatory activity, immune status, and hemoglobin concentration. Compared with simpler inflammatory ratios such as NLR, the inclusion of hemoglobin may better reflect tumor hypoxia, a mechanism considered to contribute to cisplatin resistance in bladder cancer [18,19]. Despite this rationale, the predictive value of NPLH in MIBC patients receiving NAC has not been previously evaluated.

In the present study, we assessed the association between pretreatment NPLH and pathologic response to accelerated MVAC (aMVAC) NAC in a single-center cohort of patients with MIBC. Additionally, we compared the discriminatory performance of NPLH with established inflammatory markers, including NLR and PLR.

## 2. Materials and Methods

### 2.1. Study Design and Patients

This retrospective single-center study included consecutive patients with histologically confirmed muscle-invasive bladder cancer (MIBC) treated with accelerated MVAC (aMVAC) neoadjuvant chemotherapy followed by radical cystectomy between January 2017 and December 2023. Patients with urothelial carcinoma, including cases with variant histology, were eligible for inclusion.

The inclusion criteria were clinical stage cT2–T4 N0–N3 M0 disease, age ≥ 18 years, ECOG performance status 0–2, glomerular filtration rate ≥ 50 mL/min/1.73 m^2^, and availability of complete blood count (CBC) results obtained within 28 days before NAC initiation. Patients with distant metastases, previous systemic treatment for bladder cancer, active hematologic disease, or chronic corticosteroid therapy were excluded from the analysis. All patients were qualified for aMVAC NAC per multidisciplinary team (MDT) decision and, as such, no formal screening log was maintained, as patients not selected for aMVAC by MDT received alternative treatment strategies and were not considered for inclusion.

The study was approved by the Institutional Ethics Committee (approval No. 118.0043.1.511.2024) and conducted in accordance with the Declaration of Helsinki. Due to the retrospective design of the study, the requirement for informed consent was waived.

### 2.2. Treatment Protocol

All patients received accelerated (single day) aMVAC (methotrexate 30 mg/m^2^; vinblastine 3 mg/m^2^; doxorubicin 30 mg/m^2^; cisplatin 70 mg/m^2^) with long-lasting granulocyte colony-stimulating factor (G-CSF) support on day 1 on a 14-day cycle. The planned number of cycles was 3–4, with the option to extend to 6 cycles at the treating physician’s discretion, based on response and tolerability. Radical cystectomy with standard pelvic lymphadenectomy was performed at a median of 48 days (IQR 35–62) after the last NAC cycle. Standard bilateral pelvic lymphadenectomy extending to the common iliac vessels was performed in all patients.

### 2.3. Pathologic Assessment

RC specimens were evaluated according to standard pathologic criteria. The primary outcome was pathologic complete response, defined as ypT0N0 (no residual tumor in the bladder or regional lymph nodes). Tumor regression grade was assessed using a three-tier system: TRG 1 (ypT0N0, ypTaN0, ypTisN0), TRG 2 (partial response, residual non-muscle-invasive disease), and TRG 3 (no response, residual muscle-invasive disease). All pathologic assessments were performed by dedicated uropathologists. TRG 1 (*n* = 44) encompassed ypT0N0 (*n* = 35), ypTaN0 (*n* = 6), and ypTisN0 (*n* = 3). The primary endpoint pCR was defined as ypT0N0 only.

### 2.4. Hematologic Indices

The NPLH score was calculated from the most recent CBC obtained within 28 days prior to NAC initiation, using the formula: NPLH = (absolute neutrophil count [×10^9^/L] × platelet count [×10^9^/L])/(hemoglobin [g/dL] × absolute lymphocyte count [×10^9^/L]). NLR (neutrophil count/lymphocyte count) and PLR (platelet count/lymphocyte count) were calculated from the same CBC. Patients with active infection, inflammatory conditions, or hematologic disease at the time of CBC were excluded. Active infection and inflammatory conditions were identified based on retrospective chart review of clinical notes, body temperature records, laboratory inflammatory parameters (CRP, WBC), and antibiotic use within 28 days prior to CBC collection.

### 2.5. Statistical Analysis

Continuous variables are presented as median with interquartile range (IQR), while categorical variables are reported as counts and percentages. Group comparisons were performed using the Mann–Whitney U test for continuous variables and Fisher’s exact test for categorical variables. The association between NPLH and tumor regression grade (TRG) was assessed using Spearman’s rank correlation coefficient. Differences among TRG categories were analyzed with the Kruskal–Wallis test followed by post hoc pairwise Mann–Whitney comparisons.

Receiver operating characteristic (ROC) curve analysis was used to evaluate the ability of NPLH, NLR, PLR, and hemoglobin to predict pathologic complete response (pCR). Optimal cut-off values were determined using the Youden index. Positive and negative predictive values (PPV and NPV) were subsequently calculated for the selected threshold. Formal AUC comparison was performed using the DeLong method. Bootstrap 95% CIs for AUC were estimated from 2000 resamples (seed 42). The Youden-optimal cut-off was derived in an exploratory setting from this dataset and requires external validation before clinical application.

Logistic regression analysis was performed to assess the association between NPLH and pCR. Because of its non-normal distribution, NPLH was log-transformed before inclusion in regression models. Variables included in the multivariable analysis were selected based on clinical relevance and univariate significance (*p* < 0.10) and included log-transformed NPLH and variant histology (parsimonious model, EPV = 17.5; T stage and N stage excluded as non-significant in univariate analysis and to satisfy EPV requirements). Results are presented as odds ratios (ORs) with 95% confidence intervals (CIs).

All statistical tests were two-sided, and *p*-values < 0.05 were considered statistically significant. Statistical analyses were performed using Python 3.12 with the scipy, statsmodels, and sklearn libraries.

## 3. Results

### 3.1. Patient and Treatment Characteristics

A total of 114 patients were included in the analysis. Patient and treatment characteristics are summarized in Table 1. The cohort was predominantly male (93/114, 81.6%), with a median age of 66 years (IQR 59–70; range 34–80]. The majority of patients presented with cT3 disease (55/114, 48.2%), while cT2 and cT4 accounted for 38.6% and 11.4%, respectively. Lymph node involvement at baseline was present in 15 patients (13.2%). Pure urothelial carcinoma was identified in 97 patients (85.1%); variant histology (including sarcomatoid, plasmacytoid, squamous, and glandular differentiation) was present in 17 patients (14.9%). The median number of aMVAC cycles was 4 (IQR 3–4); 71 patients (62.3%) completed at least 4 of the 6 planned cycles. Dose reduction was required in 27 patients (23.7%) and cycle delays in 40 patients (35.1%).

Pathologic complete response (ypT0N0) was achieved in 35 patients (30.7%). By TRG, 44 patients (38.6%) had TRG 1, 40 (35.1%) TRG 2, and 30 (26.3%) TRG 3. pCR rates by clinical stage were: cT2—36.4%, cT3—30.9%, and cT4—7.7%. Patients with variant histology had markedly lower pCR rates compared to pure urothelial carcinoma (5.9% vs. 35.1%). The pCR rates by number of completed aMVAC cycles were: 1–2 cycles 0% (0/5), 3 cycles 22.2% (4/18), 4 cycles 29.6% (21/71), 5–6 cycles 50.0% (10/20). Patients receiving fewer cycles typically discontinued due to toxicity rather than disease progression, which may account for the numerically lower pCR rate in this subgroup. Dose reduction was associated with lower pCR (11.1% vs. 36.8% without reduction), though this comparison was not the primary objective of this analysis.

### 3.2. NPLH and Pathologic Complete Response

**Figure 1:**
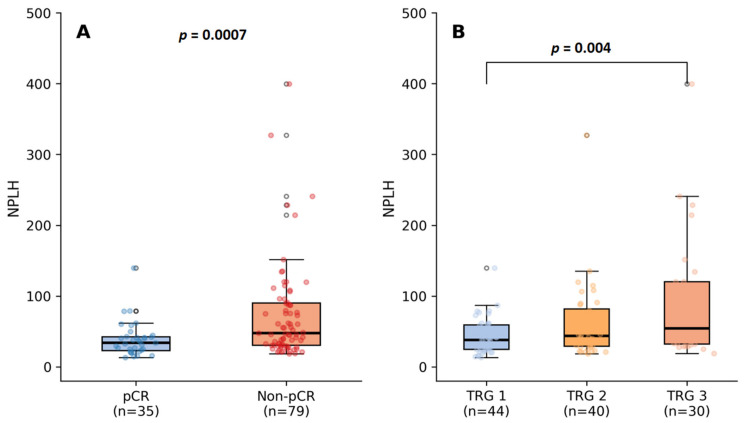
Pretreatment NPLH by pathologic response group. (**A**) NPLH distribution in patients who achieved pathologic complete response (pCR, ypT0N0) versus non-pCR. Box plots show median, interquartile range, and whiskers to 1.5 × IQR; individual data points overlaid (jitter). *p* = 0.0007, Mann–Whitney U test. (**B**) NPLH distribution across tumor regression grade (TRG) subgroups. Significant difference between TRG 1 and TRG 3 (*p* = 0.004, post-hoc Mann–Whitney). Kruskal–Wallis overall *p* = 0.010.

Pretreatment NPLH was significantly lower in patients who achieved pCR compared to non-responders (median 33.9 [IQR 23.1–42.4] vs. 47.6 [IQR 30.7–90.4]; *p* = 0.0007, Mann–Whitney U test; Table 2, Figure 1).

**Figure 2:**
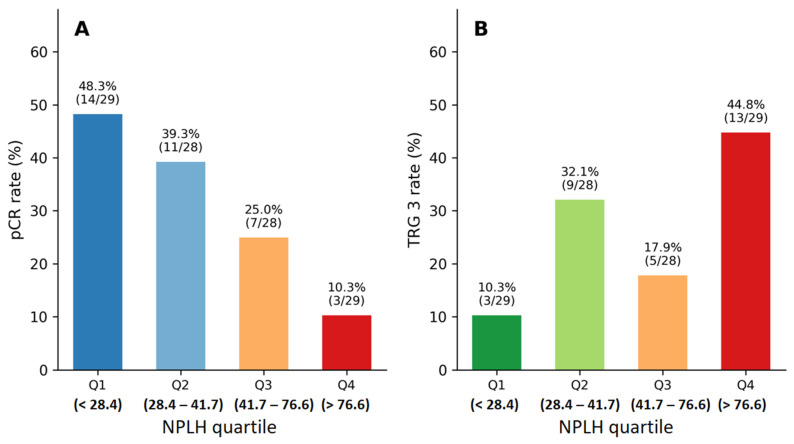
pCR and TRG 3 rates by NPLH quartile. (**A**) Pathologic complete response rate across NPLH quartiles (Q1: < 28.4; Q2: 28.4–41.7; Q3: 41.7–76.6; Q4: > 76.6). A progressive inverse gradient is observed from 48.3% in Q1 to 10.3% in Q4. (**B**) TRG 3 (no histologic response) rate across NPLH quartiles. Values above bars indicate rate (responders/total per quartile). *n* = 29 per quartile (28 for Q2 and Q3).

A monotonic inverse gradient was observed across NPLH quartiles: pCR rates were 48.3% (Q1, NPLH < 28.4), 39.3% (Q2), 25.0% (Q3), and 10.3% (Q4, NPLH > 76.6), demonstrating a near five-fold difference between the lowest and highest quartiles(Figure 2).

**Figure 3:**
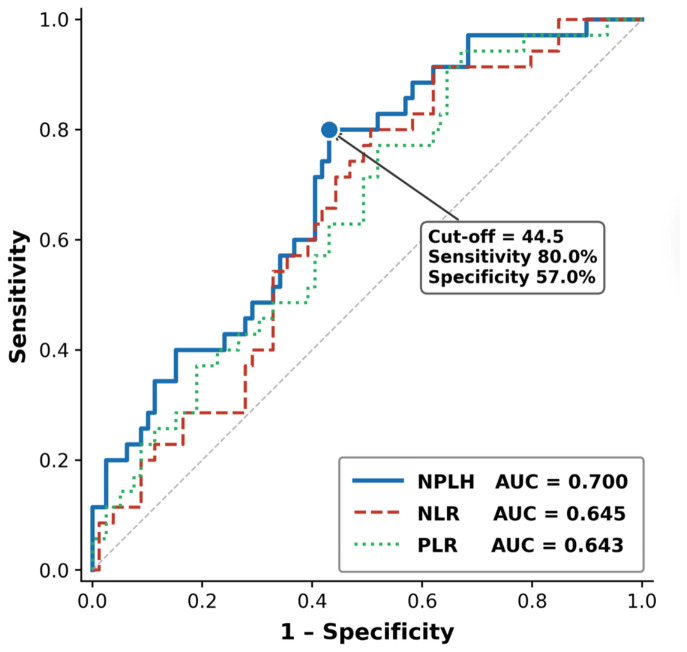
Receiver operating characteristic (ROC) curves for pCR prediction. Receiver operating characteristic (ROC) curves for NPLH, NLR, PLR, and hemoglobin for pCR prediction. The grey dotted diagonal line represents the reference line of no discrimination (AUC=0.50). Bootstrap 95% confidence intervals for AUC are reported in Table 3.

ROC curve analysis yielded an AUC of 0.700 for NPLH (Table 3, Figure 3). At the optimal Youden cut-off of 44.5, NPLH demonstrated a sensitivity of 80.0%, specificity of 57.0%, PPV of 45.2%, and NPV of 86.5%. At this threshold, patients with NPLH < 44.5 achieved pCR in 45.2% of cases compared with 13.5% in those with NPLH ≥ 44.5 (Fisher’s exact test OR 0.189, *p* = 0.0002).

### 3.3. Comparison with NLR and PLR

NPLH was numerically superior to NLR (AUC 0.645) and PLR (AUC 0.643) for pCR prediction (Table 3); however, the difference did not reach statistical significance on formal DeLong test: NPLH vs. NLR Z = 1.755, *p* = 0.079; NPLH vs. PLR Z = 1.694, *p* = 0.090. Bootstrap 95% CI: NPLH 0.700 [0.596–0.794]; NLR 0.645 [0.542–0.741]; PLR 0.643 [0.533–0.747]. The cut-off of 44.5 is exploratory and requires external validation. NLR and PLR were strongly correlated with NPLH (Spearman r = 0.864 and r = 0.849, respectively; *p* < 0.001 for both), suggesting substantial overlap in the biological information that was captured. Hemoglobin alone did not reach statistical significance as a pCR predictor (AUC 0.608, *p* = 0.066).

### 3.4. Multivariate Analysis

**Figure 4:**
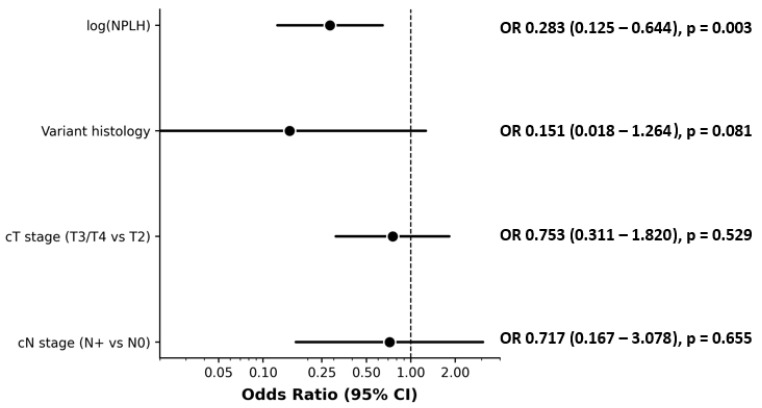
Forest plot of multivariate logistic regression for pCR prediction.

On univariate logistic regression, log-transformed NPLH (OR 0.292, 95% CI 0.131–0.652; *p* = 0.003) and variant histology (OR 0.116, 95% CI 0.015–0.911; *p* = 0.040) were significantly associated with pCR. Clinical T stage, N stage, age, NLR, and PLR did not reach statistical significance in univariate analysis. On multivariate logistic regression including log(NPLH) and variant histology (primary parsimonious model, EPV = 17.5), log(NPLH) remained the only independent predictor of pCR (OR 0.292, 95% CI 0.131–0.652; *p* = 0.003; Table 4, Figure 4). The addition of NPLH to a model including variant histology improved AUC from 0.700 (NPLH alone) to 0.731. Pseudo-R^2^ (Nagelkerke) was 0.141 (*p* < 0.001). A sensitivity analysis including cT stage and cN stage (four-covariate model, EPV = 8.75) yielded consistent results (OR log[NPLH] 0.283, 95% CI 0.125–0.644; *p* = 0.003) and is presented in Supplementary Table S1.

### 3.5. NPLH and Tumor Regression Grade

NPLH showed a positive correlation with TRG score across the full ordinal scale (Spearman r = 0.284; *p* = 0.0022), indicating progressive elevation of NPLH with worsening pathologic response. Median NPLH values were 37.9 (IQR 24.9–59.2) for TRG 1, 43.6 (IQR 29.2–81.8) for TRG 2, and 54.2 (IQR 32.4–120.3) for TRG 3. Kruskal–Wallis test indicated overall significant differences across groups (H = 9.128, *p* = 0.0104). Post-hoc comparisons revealed a significant difference between TRG 1 and TRG 3 (*p* = 0.0036), while TRG 1 vs. TRG 2 (*p* = 0.090) and TRG 2 vs. TRG 3 (*p* = 0.127) did not reach significance after pairwise testing. The TRG analysis is considered exploratory. The Spearman r = 0.284 reflects a weak-to-moderate association. The significant post-hoc contrast is confined to TRG 1 vs. TRG 3 (*p* = 0.0036); pairwise comparisons between adjacent TRG groups were non-significant and are acknowledged as such.

## 4. Discussion

In the present study, we found that pretreatment NPLH was associated with both pCR and tumor regression grade in patients treated with aMVAC. To our knowledge, this is the first study evaluating NPLH as a predictor of NAC response in MIBC.

Inflammatory markers derived from routine complete blood count testing have been widely investigated in oncology [13,14,15,16]. In bladder cancer, elevated NLR has previously been linked to worse survival after radical cystectomy and, in some studies, to lower response rates following NAC [17,18]. In our cohort, both NLR and PLR were associated with pCR, although NPLH demonstrated better discriminatory performance (AUCs of 0.700, 0.645, and 0.643, respectively). One possible explanation is the inclusion of hemoglobin within the NPLH formula, which indirectly reflects tumor oxygenation status.

The relationship between anemia, tumor hypoxia, and cisplatin resistance has been described previously. Hypoxic signaling mediated through HIF-1α promotes mechanisms associated with chemotherapy resistance, including enhanced DNA repair and autophagy [19,20]. Experimental studies in bladder cancer cell lines demonstrated reduced cisplatin-induced apoptosis under hypoxic conditions, an effect partially reversed after HIF-1α inhibition [20]. Clinically, anemia has also been associated with inferior treatment response across several solid tumors [21]. In the present study, hemoglobin alone did not significantly predict pCR, but its inclusion in the NPLH formula improved the index’s overall predictive performance.

We acknowledge that hemoglobin levels between pCR and non-pCR groups did not differ significantly in our cohort (14.2 vs. 13.6 g/dL, *p* = 0.066), which limits direct interpretation of hemoglobin as a clinically actionable proxy for tumor oxygenation. Tumor hypoxia depends on multiple factors beyond systemic hemoglobin concentration, including vascular architecture and perfusion. The contribution of hemoglobin to NPLH is therefore likely mathematical rather than directly physiological: as a denominator, it modulates the inflammatory numerator in a way that may amplify discrimination in the low-normal hemoglobin range, rather than acting as a threshold marker of anemia-driven hypoxia.

It is also possible that the lower PLR and NPLH observed in patients achieving pCR reflect underlying tumor biology. Tumors with a less pro-thrombotic microenvironment may be inherently more sensitive to chemotherapy; indeed, downregulation of the platelet activation pathway has been identified among the top five downregulated pathways in invasive MIBC [22]. Such a phenotype could be linked to the broader mitochondrial and metabolic alterations that characterize invasive MIBC [22].

In addition, neutrophils may contribute to cisplatin resistance by promoting epithelial–mesenchymal transition (EMT). Tumor-associated neutrophils release mediators such as MMP-9, TGF-β, and elastase, which can activate EMT programs. EMT is a well-recognized mechanism of chemotherapy resistance in MIBC and forms part of the molecular landscape of invasive bladder cancer [22]. Consequently, higher pretreatment neutrophil counts, as reflected by elevated NPLH values, may indicate a more EMT-prone tumor microenvironment and, therefore, a lower likelihood of achieving pCR.

The predictive performance of NPLH rests on two interacting biological axes. The inflammatory component (neutrophilia, thrombocytosis, lymphopenia) is partly driven by tumor-associated endothelial activation: endothelial cells stimulated by pro-inflammatory cytokines generate a program that sustains systemic CBC-derived inflammation [23]. On the other hand, the hemoglobin component may indirectly reflect factors potentially associated with intratumoral hypoxia: pre-existing anemia—from any cause, including cancer-related anemia of chronic disease or tumor bleeding—reduces oxygen-carrying capacity and may aggravate the hypoxic microenvironment of invasive MIBC, which already relies predominantly on anaerobic glycolysis. This hemoglobin-hypoxia axis, via HIF-1α, could promote cisplatin resistance through autophagy induction and impaired mismatch repair mechanisms [19,20]. NPLH mathematically integrates both axes in a single CBC-derived index.

NPLH shares some conceptual similarities with the HALP score (hemoglobin × albumin × lymphocytes/platelets), which has recently been proposed as a predictor of NAC response in MIBC [21]. In a previous cohort of 70 patients, lower pre-treatment HALP values were associated with a lower probability of achieving pCR. Unlike HALP, however, NPLH does not require albumin measurement and can be calculated entirely from routinely available CBC parameters. This may facilitate its implementation in daily clinical practice. Beyond inflammatory indices, molecular predictors of NAC response in MIBC—including alterations in DNA damage response genes (ERCC2, FANCC, ATM), ERBB2 mutations, and consensus molecular subtypes—have demonstrated promising discriminative value in retrospective series but require tissue-based analysis not universally available at the time of treatment decision [24]. CBC-derived indices such as NPLH offer a complementary, cost-free layer of pre-treatment stratification that does not depend on tissue availability or specialized genomic platforms.

In multivariable analysis, log-transformed NPLH remained independently associated with pCR, whereas variant histology approached statistical significance. Clinical T and N stage were not independent predictors in the final model. This may reflect the relatively limited number of patients with advanced disease in the present cohort and has also been reported in other NAC series [10,11].

The markedly lower pCR rate in patients with variant histology (5.9% vs. 35.1%) is consistent with published evidence that NAC benefit in variant histology MIBC is histotype-specific. In the GETUG-AFU V05 VESPER trial—the largest prospective evaluation of variant histology in the NAC setting—Allory et al. reported that while divergent differentiation overall was not associated with significantly altered outcomes, the presence of squamous differentiation or micropapillary subtype was associated with shorter progression-free survival [25]. The loss of significance for variant histology in the primary parsimonious multivariate model (*p* = 0.089) is likely attributable to limited statistical power (*n* = 17) rather than confounding by NPLH; the OR point estimate of 0.160 remains clinically relevant and consistent with univariate analysis. These findings reinforce the need for routine histotype characterization before NAC.

The proposed NPLH cut-off of 44.5 demonstrated high sensitivity (80.0%) but moderate specificity (57.0%) for predicting pCR. Importantly, the high negative predictive value suggests that patients with lower NPLH values are more likely to benefit from platinum-based NAC. In addition, pCR rates decreased progressively across NPLH quartiles, ranging from 48.3% in the lowest quartile to 10.3% in the highest. These findings suggest that NPLH may help identify patients at increased risk of poor response to standard chemotherapy. Whether such patients could benefit from alternative neoadjuvant strategies requires prospective validation.

The study has several limitations. First, this was a retrospective analysis from a single institution, which limits generalizability. Second, albumin measurements were unavailable, preventing direct comparison between NPLH and HALP. Third, the proposed cut-off value should be considered exploratory until externally validated. Finally, we did not evaluate long-term oncologic outcomes such as recurrence-free or overall survival. Fourth, the multivariate model was limited to two covariates to satisfy EPV requirements (EPV = 17.5); results should be considered hypothesis-generating. Fifth, survival outcomes (OS, RFS) were not assessed and will be reported in a separate analysis as follow-up matures. Sixth, Direct measurement of tumor oxygenation—including blood oxygen saturation, tissue pO_2_, or hypoxia imaging—was not available in this retrospective dataset and would be required to validate the proposed biological mechanism. Prospective studies should incorporate objective hypoxia metrics to directly test the hemoglobin-oxygenation hypothesis. Seventh, CBC-derived inflammatory indices are susceptible to confounding by non-oncologic systemic conditions; pregnancy-related states and hypertensive disorders have been shown to independently elevate neutrophil, lymphocyte, and platelet counts and derived indices such as NLR and PLR [26]. Diabetes mellitus warrants specific acknowledgement as both a potential confounder of CBC-derived indices and a biologically relevant metabolic factor: glucose-driven anaerobic metabolic reprogramming (Warburg phenotype) has been documented in invasive MIBC [22] and in urothelial carcinoma experimental models [27]. Moreover, an elevated HbA1c (≥7%) has been associated with higher recurrence rates in non-muscle invasive bladder cancer [28], suggesting that glycemic control may also influence outcomes in MIBC. Fasting glucose and HbA1c were not systematically collected, representing a meaningful limitation. Smoking, iron deficiency, cardiovascular comorbidities, and concurrent medications [29] were similarly not assessed. Structured collection of these covariates is recommended in prospective validation.

Additionally, the predominantly male cohort (81.6%), while reflective of MIBC epidemiology, may limit generalizability to female patients; sex-specific differences in T-cell exhaustion mediated by androgen receptor signaling [30] may influence lymphocyte-based inflammatory indices and should be accounted for in prospective validation studies.

Despite these limitations, the study cohort was relatively homogeneous, with all patients receiving the same NAC regimen and undergoing standardized surgical and pathological evaluation. This consistency reduces potential treatment-related confounding and supports the internal validity of the findings.

## 5. Conclusions

Pretreatment NPLH score was associated with pathologic complete response following aMVAC neoadjuvant chemotherapy in patients with muscle-invasive bladder cancer and demonstrated numerically superior performance to NLR or PLR (DeLong test *p* = 0.079). Because it is derived from routinely available complete blood count parameters, NPLH may warrant further evaluation as a simple and inexpensive exploratory biomarker for pretreatment risk stratification in MIBC. Further prospective studies are needed to validate these findings and to determine whether NPLH could support the selection of patients for alternative neoadjuvant treatment strategies.

## Figures and Tables

**Table 1:** Patient, treatment, and response characteristics.

Characteristic	All Patients (*n* = 114)	
Age, years—median (IQR)	66 (59–70)	range 34–80
Sex, *n* (%)		
Male	93 (81.6%)	
Female	21 (18.4%)	
Clinical T stage, *n* (%)		
cT2	44 (38.6%)	
cT3	55 (48.2%)	
cT4	13 (11.4%)	
Clinical N stage, *n* (%)		
cN0	97 (85.1%)	
cN+	15 (13.2%)	
cNx	2 (1.8%)	
Histologic variant, *n* (%)		
Pure urothelial	97 (85.1%)	
Variant histology	17 (14.9%)	
Chemotherapy cycles, median (IQR)	4 (3–4)	
Dose reduction, *n* (%)	27 (23.7%)	
Cycle delay, *n* (%)	40 (35.1%)	
Time from last NAC cycle to RC, days—median (IQR)	48 (35–62)	range 17–122
Pathologic response, *n* (%)		
pCR (ypT0N0)	35 (30.7%)	
TRG 1	44 (38.6%)	
TRG 2	40 (35.1%)	
TRG 3	30 (26.3%)	
Pretreatment laboratory parameters		
Neutrophils, ×10^9^/L—median (IQR)	4.54 (3.56–5.85)	
Lymphocytes, ×10^9^/L—median (IQR)	1.94 (1.60–2.48)	
Hemoglobin, g/dL—median (IQR)	13.90 (12.70–14.70)	
Platelets, ×10^9^/L—median (IQR)	266.5 (225.0–326.0)	
NLR—median (IQR)	2.23 (1.69–3.08)	
PLR—median (IQR)	127.9 (98.9–196.4)	
NPLH—median (IQR)	41.7 (28.4–76.6)	range 13.2–882.0

No variables had missing data. cNx: clinically indeterminate nodal status (*n* = 2, excluded from N0/N+ classification). aMVAC, accelerated MVAC; IQR, interquartile range; pCR, pathologic complete response (ypT0N0); TRG, tumor regression grade.

**Table 2:** Pretreatment hematologic indices by pathologic complete response status.

	pCR (*n* = 35)	Non-pCR (*n* = 79)	*p* Value
NPLH—median (IQR)	33.9 (23.1–42.4)	47.6 (30.7–90.4)	0.0007
NLR—median (IQR)	2.01 (1.56–2.55)	2.44 (1.74–3.40)	0.0138
PLR—median (IQR)	119.0 (87.2–171.4)	145.8 (108.0–213.3)	0.0150
Hemoglobin, g/dL—median (IQR)	14.2 (13.1–15.2)	13.6 (12.3–14.5)	0.0657

*p* values from Mann–Whitney U test. NLR, neutrophil-to-lymphocyte ratio; NPLH, neutrophils × platelets/hemoglobin × lymphocytes; PLR, platelet-to-lymphocyte ratio.

**Table 3:** ROC curve analysis: performance of hematologic indices for pCR prediction.

Marker	AUC (95% CI)	Optimal Cut-Off	Sensitivity	Specificity	*p*-Value *
NPLH	0.700 (0.596–0.794)	44.5 †	80.0%	57.0%	0.0007
NLR	0.645 (0.542–0.741)	2.86	91.4%	38.0%	0.0138
PLR	0.643 (0.533–0.747)	196.6	94.3%	32.9%	0.0094
Hgb	0.608 (0.498–0.715)	13.8 g/dL	68.6%	51.9%	0.0657

* Mann–Whitney U test p value for comparison between pCR and non-pCR groups. AUC 95% confidence intervals estimated by bootstrap (2000 resamples, seed 42). DeLong test for AUC comparisons: NPLH vs. NLR Z = 1.755, *p* = 0.079 (NS); NPLH vs. PLR Z = 1.694, *p* = 0.090 (NS). † Optimal cut-off determined by Youden index; derived from this dataset and requires external validation before clinical application. AUC, area under the curve; NLR, neutrophil-to-lymphocyte ratio; NPLH, neutrophils × platelets/hemoglobin × lymphocytes; PLR, platelet-to-lymphocyte ratio, Hgb—hemoglobin.

**Table 4:** Uni- and multivariate logistic regression for prediction of pCR.

Variable	OR	95% CI	*p* Value
Univariate analysis			
log(NPLH)	0.275	0.127–0.594	0.001
Variant histology	0.116	0.015–0.911	0.040
log(NLR)	0.294	0.114–0.758	0.011
log(PLR)	0.260	0.094–0.719	0.009
cT stage (T3/T4 vs. T2)	0.614	0.274–1.374	0.235
cN stage (N+ vs. N0)	0.523	0.138–1.986	0.341
Age	1.004	0.958–1.051	0.874
Multivariate analysis			
log(NPLH)	0.292	0.131–0.652	0.003
Variant histology	0.160	0.019–1.319	0.089

CI, confidence interval; OR, odds ratio. NPLH was log-transformed prior to analysis. Primary parsimonious model: log(NPLH) + variant histology; EPV = 17.5; Pseudo-R^2^ (Nagelkerke) = 0.141; overall model *p* < 0.001. The four-covariate sensitivity analysis (+ cT stage + cN stage, EPV = 8.75) is presented in Supplementary Table S1.

## Data Availability

The data supporting the findings of this study are available from the corresponding author (P.J.W.) upon reasonable request, subject to institutional governance and ethical restrictions.

## Supplementary Materials

The following supporting information can be downloaded at: https://www.mdpi.com/article/10.3390/cancers18132046/s1, Table S1: Four-covariate sensitivity analysis: multivariate logistic regression for prediction of pathologic complete response (pCR).

## Abbreviations

The following abbreviations are used in this manuscript:

aMVAC	Accelerated MVAC (methotrexate, vinblastine, doxorubicin, cisplatin)
AUC	Area under the curve
CI	Confidence interval
CBC	Complete blood count
ECOG	Eastern Cooperative Oncology Group
IQR	Interquartile range
MIBC	Muscle-invasive bladder cancer
NLR	Neutrophil-to-lymphocyte ratio
NPLH	Neutrophil-to-platelet-hemoglobin-lymphocyte ratio
OR	Odds ratio
pCR	Pathological complete response
PLR	Platelet-to-lymphocyte ratio
PPV	Positive predictive value
RC	Radical cystectomy
ROC	Receiver operating characteristic
TRG	Tumor regression grade
