# Supplementary material for: Pretreatment NPLH as a Potential Predictor of Pathologic Complete Response to Accelerated MVAC Neoadjuvant Chemotherapy in Muscle-Invasive Bladder Cancer: Comparison with NLR and PLR
Source: Cancers (Basel). 2026 Jun 24;18(13):2046. doi: 10.3390/cancers18132046 (PMC13359953; doi:10.3390/cancers18132046)
Supplement: Supplementary file 1 [file cancers-18-02046-s001.zip › cancers-4361812-supplementary.pdf]

Supplementary Material

Table S1. Full logistic regression analysis: univariate results and four-covariate sensitivity multivariate model for prediction of pathologic complete response (pCR).

Variable	OR	95% CI	p value
Univariate analysis			
log(NPLH)	0.292	0.131–0.652	0.003
Variant histology	0.116	0.015–0.911	0.040
cT stage (T3/T4 vs T2)	0.614	0.274–1.374	0.235
cN stage (N+ vs N0)	0.523	0.138–1.986	0.341
Age (per year)	1.004	0.958–1.051	0.874
log(NLR)	0.294	0.114–0.758	0.011
log(PLR)	0.260	0.094–0.719	0.009
Multivariate analysis — four-covariate sensitivity model			
log(NPLH)	0.283	0.125–0.644	0.003
Variant histology	0.151	0.018–1.264	0.081
cT stage (T3/T4 vs T2)	0.753	0.311–1.820	0.529
cN stage (N+ vs N0)	0.717	0.167–3.078	0.655

CI, confidence interval; cN, clinical nodal stage; cT, clinical tumour stage; EPV, events per variable; NLR, neutrophil-to-lymphocyte ratio; NPLH, (neutrophils × platelets) / (hemoglobin × lymphocytes); OR, odds ratio; PLR, platelet-to-lymphocyte ratio.

NPLH, NLR, and PLR were log-transformed prior to analysis. The four-covariate sensitivity multivariate model includes log(NPLH) + variant histology + cT stage + cN stage; EPV=8.75 (35 events / 4 covariates); Pseudo-R^2 (Nagelkerke)=0.141; overall model $p<0.001$. Results are consistent with the primary parsimonious two-covariate model (log[NPLH] + variant histology, EPV=17.5; OR log[NPLH] 0.292 [0.131–0.652], p=0.003) reported in Table 4 of the main manuscript.
